# Knobloch Syndrome Associated with Novel *COL18A1* Variants in Chinese Population

**DOI:** 10.3390/genes12101512

**Published:** 2021-09-26

**Authors:** Songshan Li, You Wang, Limei Sun, Wenjia Yan, Li Huang, Zhaotian Zhang, Ting Zhang, Xiaoyan Ding

**Affiliations:** State Key Laboratory of Ophthalmology, Zhongshan Ophthalmic Center, Sun Yat-sen University, Guangzhou 510000, China; lisongshan@gzzoc.com (S.L.); wangy998@mail2.sysu.edu.cn (Y.W.); sunlimei@gzzoc.com (L.S.); wenj.yan@outlook.com (W.Y.); huangli@gzzoc.com (L.H.); zhangzhaotian@gzzoc.com (Z.Z.); zhangting@gzzoc.com (T.Z.)

**Keywords:** Knobloch syndrome, *COL18A1*, macular dysplasia, whole exon sequencing

## Abstract

Knobloch syndrome is an inherited disorder characterized by high myopia, retinal detachment, and occipital defects. Disease-causing mutations have been identified in the *COL18A1* gene. This study aimed to investigate novel variants of *COL18A1* in Knobloch syndrome and describe the associated phenotypes in Chinese patients. We reported six patients with Knobloch syndrome from four unrelated families in whom we identified five novel *COL18A1* mutations. Clinical examination showed that all probands presented with high myopia, chorioretinal atrophy, and macular defects; one exhibited rhegmatogenous retinal detachment in one eye. Occipital defects were detected in one patient.

## 1. Introduction

Knobloch syndrome (KS, MIM# 267750) is a rare syndromic autosomal recessive disorder, characterized by eye abnormalities and occipital defects. The ocular manifestations include high myopia, retinal detachment, congenital cataracts, lens subluxation, vitreoretinal degeneration, and retinitis pigmentosa-like features [1,2,3]. Numerous extraocular abnormalities have been reported, including occipital defects, encephalocele, hydrocephalus, polymicrogyria, bifid ureter, dextrocardia, and seizures [4,5,6]. 

The most common disease-causing molecular defects are mutations of the *COL18A1* gene (OMIM# 120328), located on chromosome 21q22.3. The gene comprises of 41 exons and encodes the collagen alpha-1 (XVIII) chain, a basement membrane constituent highly expressed throughout the eye. Collagen XVIII has an essential role in ocular development, including angiogenesis and structural maintenance [7,8]. To date, there have been 48 genetically confirmed KS families of various ethnicities; nevertheless, KS is rarely reported in east Asian populations. Only one Chinese family was reported to have a homozygous *COL18A1* mutation [9]. However, the clinical appearance in this family was limited, due to the opacity of the optical media (Supplementary Table S1, *COL18A1* mutation of patients with KS) [3,10,11,12,13,14,15,16,17,18,19,20,21].

Here, we present the extensive study of six affected individuals with KS, from four unrelated Chinese families, using comprehensive multimodal imaging and genetic testing, in which we identified five novel pathogenic mutations of *COL18A1*. This study could help to further explore the genotypic and phenotypic spectrum of KS.

## 2. Materials and Methods

Five KS patients, from four unrelated families, were referred to Zhongshan ophthalmic center and included in this study. The diagnoses of KS were based on characteristic clinical features and confirmation by identification of pathogenic *COL18A1* variants. This study was conducted following the tenets of the Declaration of Helsinki and was approved by the institutional review board of Zhongshan Ophthalmic Center, Sun Yat-sen University. Informed written consent was obtained from the patients or their legal guardians.

Clinical information, including age, sex, history, pedigree, symptoms, and clinical diagnosis, were recorded. Detailed ophthalmic examinations included the best corrected visual acuity (BCVA), intraocular pressure, refractive errors, axial length (IoL Master, Carl Zeiss, Meditec, Jena, Germany), slit lamp biomicroscopy, wild field scanning laser ophthalmoscope (Optomap 200Tx, Optos pls, Dunfermline, UK), or RetCam imaging (RetCam, Clarity Medical Systems Inc., Pleasanton, CA, USA). Full-field flicker ERGs were recorded using the RETeval system (LKC Technologies, Gaithersburg, MD, USA); ISCEV standard flicker parameters were followed. Four patients underwent magnetic resonance imaging of the brain.

Genomic DNA of the proband and available family members was extracted from peripheral blood using the TIANamp Blood DNA Kit (DP348-03, Tiangen Biotech, Beijing, China), as instructed by the manufacturer. The quantity and quality of DNA were verified by using NanoDrop. Whole-exome sequencing (WES) was performed for four probands, and identified mutations were validated using Sanger sequencing of the family members. The Human Gene Mutation Database, Genome Aggregation Database, and Exome Aggregation Consortium were used to identify the reported pathogenic variants. Online algorithms, including MutationTaster, sorting intolerant from tolerant (SIFT), and Polymorphism Phenotyping v2 (Polyphen2), were used to evaluate the pathogenicity of missense mutations.

## 3. Results

### 3.1. Clinical Features

**Figure 1:**
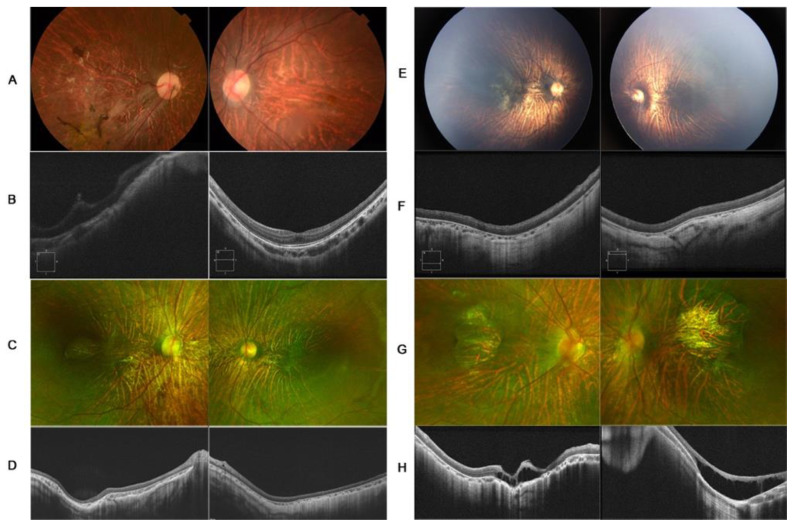
Fundus photographs and macular coherence tomography (OCT) images in patient 1 (**A**,**B**), patient 2 (**C**,**D**), patient 3 (**E**,**F**), and patient 4 (**G**,**H**). (**A**) Fundus photography showed tessellated fundus in patient 1. The increased pigmentation in the right eye was induced by a history of retinal detachment. (**B**) OCT revealed mild hypoplasia in the left eye. (**C**) SLO showed tessellated fundus appearance, prominent choroidal, and punched-out macular in patient 2. (**D**) OCT revealed undetected macular and posterior staphyloma. (**E**) Fundus images showed tessellated fundus and prominent choroidal in patient 3. (**F**) OCT revealed undetected macular and posterior staphyloma. (**G**) Fundus images revealed tessellated fundus appearance and well-bounded macular RPE atrophic changes in patient 4. (**H**) OCT showed macular retinoschisis and posterior staphyloma.

**Figure 2:**
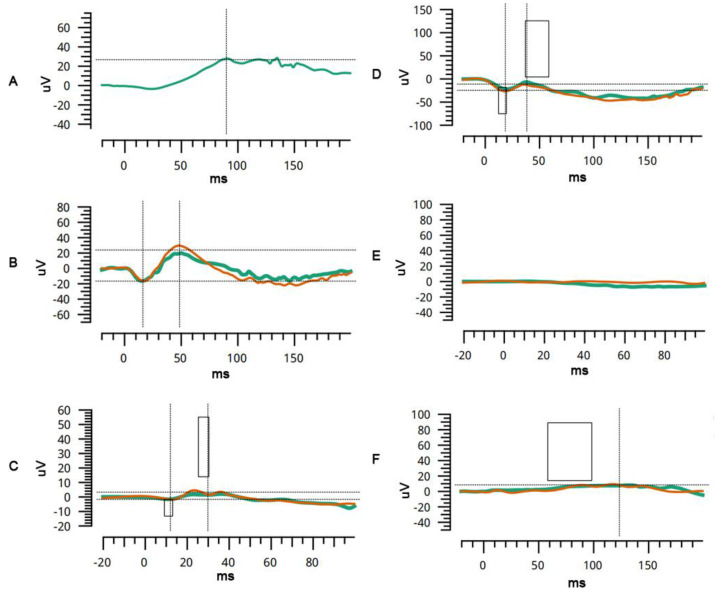
Characteristic ERG findings in patients with Knobloch Syndrome. In patient 3, rod-specific ERG (dark adapted 0.01, (**A**)) and bright flash (dark adapted 3, (**B**)) is mildly subnormal. Single flash (light adapted 3) showed severely diminished B-wave, indicating a major cone function impairment. (**C**) In patient 4, rod-specific ERG showed severe diminished on rod-specific ERG (**D**) and bright flash; (**E**) cone single flash was almost extinguished, (**F**) These results indicated the loss of function in both cone and rod photoreceptors.

Homozygous or compound heterozygous mutations were identified in six patients diagnosed with KS, from four unrelated families. The clinical manifestations are shown in Figure 1 and Figure 2. The clinical data are summarized in Table 1.

#### 3.1.1. Patient 1

A 7-year-old girl, with a history of rhegmatogenous retinal detachment and pars plana vitrectomy in her right eye at 4 years of age, was referred to Zhongshan Ophthalmology Center with symptoms of blurred vision in the left eye. The BCVA of the proband was hand move/before eye OD and 0.3 logMAR OS. The axial lengths of the right and left eyes were 27.08 mm and 26.92 mm, respectively, with spherical equivalents of −15.0D OD and −14.25D OS. The anterior segment was unremarkable. Fundus examination revealed clumped pigmentary deposition in the right eye and tessellated funds in the left eye. OCT was mildly abnormal in her left eye, with slightly macular hypoplasia. The retina remained attached in her right eye (Figure 1A,B). A compound heterozygous mutation of the *COL18A1* gene was detected, c.4054_4055delCT (p.Leu1352Valfs*72) in exon39 and c.2992G>A (p.Gly998Arg) in exon 28. The same mutations were identified in her two brothers. Her 11-year-old brother had a diagnosis of congenital cataracts in both eyes at 1 year of age and underwent cataract surgery. Retinal detachment in the left eye was noted at 10 years of age, and pars plana vitrectomy was performed as treatment. The BCVA was hand move/30 cm OD and no light perception OS. Examination revealed a longstanding retinal detachment in his right eye and corneal leukoma of his left eye. Her 1-year-old brother underwent a fundus examination by RetCam for poor vision and nystagmus, since 3 months of age, revealing tessellated fundus and macular dysplasia of both eyes (Supplementary Figure S1A–D).

#### 3.1.2. Patient 2

A 3-year-old girl, from a non-consanguineous family, was referred to our hospital for high myopia and low vision detected on a routine examination. Her family history was unremarkable. Examination revealed horizontal conjugate nystagmus in both eyes. The spherical equivalent was −8.25 diopters in the right eye and −8.50 diopters in the left eye, with axial lengths of 25.16 mm and 25.19 mm, respectively. The intraocular pressure and anterior segment were unremarkable. Dilated fundus examination revealed empty vitreous, tessellate fundus, retinal pigmental changes, and punched-out macular in both eyes. The features in OCT included outer segment retina atrophy, significant thinning of the retina and choroid, macular hypoplasia, and posterior staphyloma. A homozygous *COL18A1* variant, c.3810dup (p.Val1271Arg fs*27), was identified by WES. Sanger sequencing showed that both parents and her older sister were heterozygous carriers (Figure 1C,D).

#### 3.1.3. Patient 3

A 6-month-old boy, from a non-consanguineous family, presented with nystagmus since birth. The family history was unremarkable. The patient exhibited horizontal conjugate nystagmus in both eyes. Examination revealed axial lengths of 23.55 mm OD and 24.00 mm OS, with spherical equivalents of −9D OD and −10D OS. The anterior segment was unremarkable. Fundus examination by RetCam revealed widespread pigmentary changes, chorioretinal atrophy, and macular hypoplasia in both eyes. SD-OCT confirmed macular hypoplasia with the non-identifiable foveal pits. A poorly laminated retinal layer and absence of IZ-EZ (interdigitation zone and ellipsoid zone) were also identified. Flash electroretinogram showed a slightly decreased amplitude under scotopic conditions and dramatically decreased amplitude under photopic conditions. Genetic testing revealed a homozygous mutation for the *COL18A1* gene, c.3364_3372 delGGCCCCCCAinsC. Sanger sequencing showed that both parents and her older sister were heterozygous carriers (Figure 1E,F and Figure 2A–C).

#### 3.1.4. Patient 4

A 2-year-old boy, born from a non-consanguineous family, presented with poor vision for six months. His personal history and family history were unremarkable. Cycloplegic refraction showed high myopia: −12.5 and −12 spherical equivalent diopters for the right and left eye, respectively. The axial lengths of the right and left eyes were 25.67 mm and 25.38 mm, respectively. The anterior segment was unremarkable. Fundus examination revealed the tessellate fundus, posterior scleral staphyloma, and macular pseudocolobomas. OCT revealed apparent scleral staphyloma, chorioretinal atrophy, macular retinoschisis, and poor retinal lamination. Flash electroretinogram showed an almost extinguished pattern under both scotopic and photopic conditions, which indicated the loss of function of cone and rod photoreceptors (Figure 1G,H and Figure 2D–F). A focal occipital skin defect was observed on the occipital scalp (Supplementary Figure S1). MRI revealed a normal occipital skull. A compound heterozygous mutation was identified by genetic analysis: c.1999C>T (p.Arg667Ter) and c.3213dup(p.Gly1072ArgfsTer9). Sanger sequencing showed that both parents were heterozygous carriers.

### 3.2. Genetic Finding

**Figure 3:**
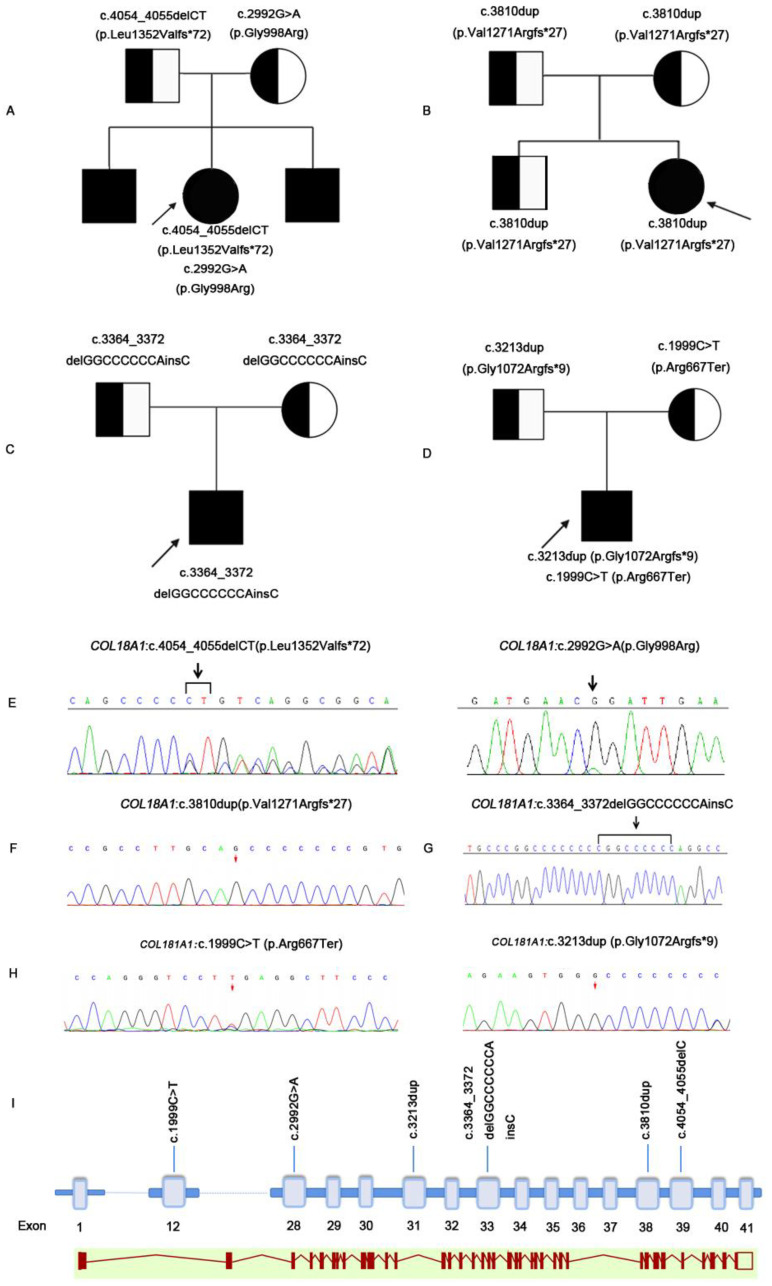
Pedigrees and genetical changes of families affected by Knobloch Syndrome. Two homozygous and two complex heterozygous mutations in *COL18A1* were identified. Patient 1: (**A**,**E**). Patient 2: (**B**,**F**). Patient 3: (**C**,**G**). Patient 4: (**D**,**H**). The exon–intron structure and the variants in this study were shown on the scheme (**I**).

Pedigree graphs of the four families are shown in Figure 2. Six *COL18A1* pathogenic mutations were detected in four families. The coding impact of one of the mutations was missense: c.2992G>A(p.Gly998Arg); one of the mutations was nonsense: c.1999C>T(p.Arg667Ter); and four of the mutations were frameshift: c.4054_4055delC(p.Leu1352ValTer72), c.3810dup(p.Val1271ArgfsTer27), c.3364_3372delGGCCCCCCAinsC(p.Gly1122ArgfsTer142), and c.3213dup(Gly1072ArgfsTer9). The mutated sequence in the identified pathogenic variants was highly conserved in the homologs of *COL18A1* (Supplementary Figure S1E). Of the six mutations, five (83.3%) were novel (Table 2 and Figure 3).

## 4. Discussion

Considering the rarity of this disease, the spectrum of the genetic variability remains to be discovered. Since being shown to be associated with KS in 2000 [22], *COL18A1* remains the only gene to be definitively, causally related to the disease. Although *ADAMTS18* was once reported to be disease-causing in KS, a splicing mutation of *COL18A1* was later reported in the same patients. To date, 22 *COL18A1* mutations have been reported to be likely pathogenic [13]. In the present study, we identified five new mutations of *COL18A1*, in four distinct KS families. Three of these were frameshift mutations, one was missense mutation, and one was nonsense mutation. The mutations were compound heterozygous in two families and homozygous in the other two families for the respective sites. Our results accord with previous studies, in which the majority of *COL18A1* variants (5/6, 83.3%) that led to KS were protein-truncating variants [13]. In this study, one missense mutation has been detected. Although patient 1, with the heterozygous missense mutation and frameshift mutation, presented as a mild type. Considering the clinically heterogeneous of this disease, the genotype–phenotype correlation still needs further exploration.

Since being first reported by Knobloch in 1971, 48 genetically confirmed KS families have been reported. KS is classically defined as a triad of high myopia, retinal detachment, and occipital defect. The ophthalmic features are diverse, on account of the severity and course of the disease. In the present study, all patients presented with early-onset high myopia, with refractive diopters higher than −8 D. All patients showed retina changes, including tessellated fundus and macular abnormalities. Two patients had retinal detachment, due to the peripheral retinal tear before age 10. Because of the tendency of retinal detachment, prophylactic laser photocoagulation might be considered to improve visual outcomes.

Interestingly, occipital change was noticed in only one patient. Our results suggest that the eye phenotype alone can be pathognomonic. Children with early-onset high myopia, presenting with retinal changes at a very young age, should be suspected of having KS and undergo genetic testing, even without occipital changes.

In addition to high myopia and retinal detachment, other ocular manifestations have been described as characteristic features of KS. Hull et al. [13] reported cone–rod dysfunction, revealed by electrophysiologic examination. Chorioretinal atrophy and colobomas have also been reported [17,23]. In the present study, we observed macular abnormalities in all KS patients, from mild macular hypoplasia to completely undetectable macular and retinoschisis. One proband had macular hypoplasia grade I; two probands had posterior staphyloma and macular hypoplasia grade IV; one proband had macular retinoschisis and macular hypoplasia grade IV. Although posterior staphyloma and macular atrophy are not specific and could occur secondary to high myopia, secondary changes in high myopia require long periods to occur. Thus, we suggest that macular changes, especially macular hypoplasia, could be a primary and key feature of KS.

The limitations of our study are as follows: (1) the sample size was limited to six, due to the rarity of KS; (2) all patients were recruited from the pediatric ophthalmology department of Zhongshan ophthalmic center, which might introduce selection bias; and (3) although characteristic clinical manifestations were observed in all patients, novel mutations will be further confirmed in future studies.

## 5. Conclusions

In conclusion, we identified novel *COL18A1* mutations as causes of KS. Considering the variable manifestations, the diagnosis of KS might be considered in any patient with early-onset high myopia and macular abnormalities. 

## Figures and Tables

**Table 1:** Clinical features of the KS patients.

Pedigree/ID/ Sex/Age, Years	Symptoms	BCVA	Axial Length, mm	Spherical Equivalent, Diopter	Anterior Segment	Fundus	Macular	Thinning Choroid	ERG	Systemic Abnormalities
A/DX0458/ F/7	Blurred vision	HM/before eye	27.08	−15	normal	clumped pigmentary deposition (post-surgery)	NA	NA	NA	-
		0.3 logMAR	26.92	−14.25	normal	tessellated	macular dysplasia grade I	+	reduced amplitudes	
A// M/11	Vision loss	HM/30 cm	NA	NA	aphakia (post-surgery)	Retinal detachment	NA	NA	NA	-
		NLP	NA	NA	cornea leukoma, aphakia (post-surgery)	NA	NA	NA	NA	
A/ M/1	nystagmus	NA	23.85	NA	normal	tessellated	punched-out, macular dysplasia	NA	NA	-
		NA	23.70	NA	normal	tessellated	punched-out, macular dysplasia	NA	NA	
B// F/3	Low vision	FC/50 cm	25.16	−8.25	normal	tessellated	punched-out, macular dysplasia grade IV	+	almost extinguished	-
		FC/50 cm	25.19	−8.5	normal	tessellated	punched-out, macular dysplasia grade IV	+	almost extinguished	
C/XDW1045/ M/0.5	nystagmus	NA	23.55	−9	normal	tessellated	macular dysplasia grade IV	+	reduced amplitudes	-
		NA	24.00	−10	normal	tessellated	macular dysplasia grade IV	+	reduced amplitudes	
D/AP34796/ M/2	low vision	HM/50 cm	25.67	−12.5,	normal	tessellated	punched-out, retinoschisis	+	almost extinguished	Focal occipital skin defect
		HM/50 cm	25.38	−12	normal	tessellated	punched-out, retinoschisis	+	almost extinguished	

HM: hand motion, NLP: no light perception, FC: finger counting, NA: not available, +: positive, -: negative.

**Table 2:** Clinical features of the KS patients.

Pedigree	Chr-ID	Nucleotide	Zygosity	Type	Exon	Protein Changes	Allele Frequency(gnomAD)	Polyphen2	Mutation Taster	SIFT	Cadd Scores	Source
A	chr21: 46930004	NM_030582.4:c.4054_4055delC	heterozygous	frameshift	39	p.Leu1352ValTer72	NA	/	/	/	26.5	Novel
	chr21: 46915290	NM_030582.4:c.2992G>A	heterozygous	Missense	28	p.Gly998Arg	NA	Damaging	Damaging	Damaging	25.4	Novel
B	chr21: 46929293	NM_030582.4:c.3810dup	homozygous	frameshift	38	p.Val1271ArgfsTer27	NA	/	/	/	33	Novel
C	chr21: 46924425	NM_030582.4:c.3364_3372 delGGCCCCCCAinsC	homozygous	frameshift	33	p.Gly1122ArgfsTer142	NA	/	/	/	33	Novel
D	chr21: 46900620	NM_030582.4:c.1999C>T	heterozygous	nonsense	12	p.Arg667Ter	0.000056	/	/	/	36	Novel
	chr21: 469175 58	NM_030582.4:c.3213dup	heterozygous	frameshift	31	p.Gly1072ArgfsTer9	NA	/	/	/	33	Database

## Data Availability

All raw data used during the study are available from the corresponding author by request.

## Supplementary Materials

The following are available online at https://www.mdpi.com/article/10.3390/genes12101512/s1, Figure S1: The clinical feature and amino acid sequences of two brother of family A and skin defect of proband of family D. The fundus photograph or anterior segmental photography of two brothers from family A (A–D) and the occipital skin defect of proband D (F). The old brother presented with visual loss and uniocular blindness. The fundus images of right eye showed tessellated changes and retinal detachment occurred soon after presence (A). The anterior segmental image showed corneal leukoma of left eye (B). The fundus photograph of younger brother showed tessellated fundus appearance and macular dysplasia (C, D). The result of amino acid sequences of c.2992g>A from nine species reported in the mutation taster is shown (E). A focal occipital skin defect was observed on the occipital scalp in the proband of family D (F). Table S1: *COL18A1* mutation of patients with KS.
