# Supplementary material for: Knobloch Syndrome Associated with Novel COL18A1 Variants in Chinese Population
Source: Genes (Basel). 2021 Sep 26;12(10):1512. doi: 10.3390/genes12101512 (PMC8536191; doi:10.3390/genes12101512)
Supplement: Supplementary file 1 [file genes-12-01512-s001.zip › legend-S.pdf]

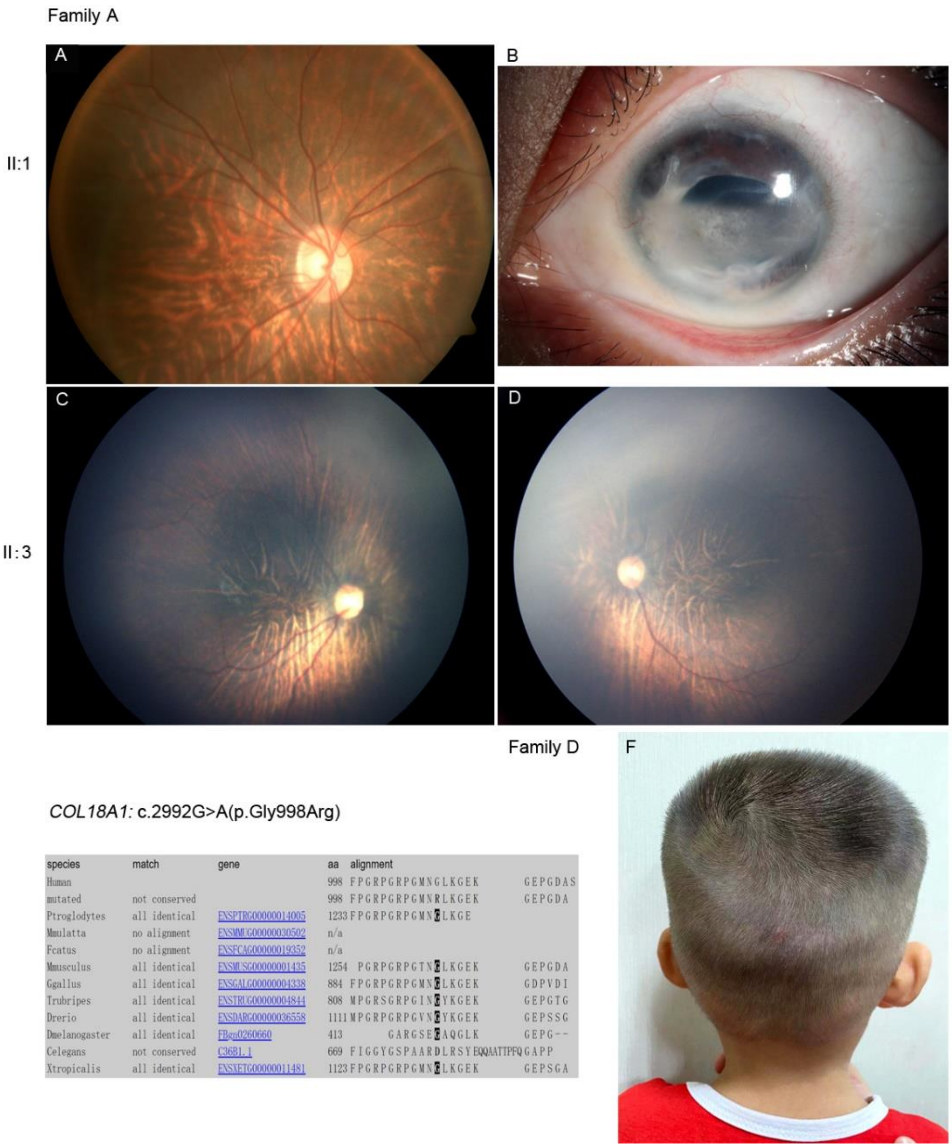

species	match	gene	aa	alignment
Human			998	FPGRPGRPGMNGLKGEK GEPGDAS
mutated	not conserved		998	FPGRPGRPGMNRLKGEK GEPGDA
Ptroglodytes	all identical	ENSPTRG00000014005	1233	FPGRPGRPGMNGLKGE
Mmulatta	no alignment	ENSMMUG00000030502	n/a	
Fcatus	no alignment	ENSFCAG00000019352	n/a	
Mmusculus	all identical	ENSMUSG00000001435	1254	PGRPGRPGTNGLKGEK GEPGDA
Ggallus	all identical	ENSGALG00000004338	884	FPGRPGRPGMNGLKGEK GDPVDI
Trubripes	all identical	ENSTRUG00000004844	808	MPGRSGRPGINGYKGEK GEPGTG
Drerio	all identical	ENSDARG00000036558	1111	MPGRPGRPGVNGYKGEK GEPSSG
Dmelanogaster	all identical	FBgn0260660	413	GARGSEGAQGLK GEPG--
Celegans	not conserved	C36B1.1	669	FIGGYGSPAARDLRSYEQQAATTPFQGAPP
Xtropicalis	all identical	ENSXETG00000011481	1123	FPGRPGRPGMNGLKGEK GEPSGA

Figure S1:The clinical feature and amino acid sequences of two brother of family A and skin defect of proband of family D

The fundus photograph or anterior segmental photography of two brothers from family A (A-D) and the occipital skin defect of proband D (F). The old brother presented with visual loss and uniocular blindness. The fundus images of right eye showed tessellated changes and retinal detachment occurred soon after presence (A). The anterior segmental image showed corneal leukoma of left eye (B). The fundus photograph of younger brother showed tessellated fundus appearance and macular dysplasia (C, D). The result of amino acid sequences of c.2992g>A from nine species reported in the mutation taster is shown (E). A focal occipital skin defect was observed on the occipital scalp in the proband of family D (F).
