# Supplementary material for: Knobloch Syndrome Associated with Novel COL18A1 Variants in Chinese Population
Source: Genes (Basel). 2021 Sep 26;12(10):1512. doi: 10.3390/genes12101512 (PMC8536191; doi:10.3390/genes12101512)
Supplement: Supplementary file 1 [file genes-12-01512-s001.zip › Table-S.pdf]

Supplementary Table S1: *COL18A1 m*utation in patients diagnosed with KS

Mutations	Type	Race or nationality	Gender	Ocular findings				Systemic Features	Reference
				myopia	Anterior segment features	Posterior segment features	Others		
IVS1-2A>T	Hom	Brazilian	F	Y	NA	Vitreo-retinal degeneration	None	NA	Suzuki OT et al.[1]
c.355del	Hom	Saudi	M	Y	Ectopia lentis	Vitreo-retinal atrophy	None	Encephalocele, epilepsy and mental delay	Khan AO et al.[2]
c.355del	Hom	NA	F	Y	NA	RD related to macular hole	Nystagmus	NA	Alsulaiman SM et al.[3]
c.1238_1239ins c.3514_3515del	Compound het	North American	M	Y	NA	Vitreo-retinal degeneration, macular degeneration, retinal detachment	None	NA	Suzuki OT et al.[1]
c.1610del	Hom	Belgian	M	Y	Lens dislocation	Macular pseudocolobomas and chorioretinal atrophy	Nystagmus	Occipital bone abnormality	Balikova I et al.[4]
c.1785_1786delinsA	Hom	Saudi	M	Y	Normal	Vitreo-retinal atrophy	Exotropia	Focal hair defect	Khan AO et al.[2]

c.2437-2A>G c.3213delC	Compound het	NA	M	Y	Persistent pupillary membrane	Chorioretinal atrophy	None	Hypermobile joints	Hull S et al.[5]
c.2658dupC	Het	NA	F	Y	cortical lens opacity	Choroiretinal atrophy	None	Occipital meningocele	Hull S et al.[5]
c.2673delC	Hom	NA	F	Y	NA	RD related to macular hole	None	NA	Alsulaiman et al.[3]
c.2743C>T	Hom	Saudi	M	Y	Ectopia lentis	Vitreo-retinal atrophy	Nystagmus	Focal hair defect	Khan AO et al.[2]
c.2797C>T c.3213dupC	Compound het	NA	M	NA	NA	NA	Nystagmus	Cysts	Thau A et al.[6]
c.2960_2969dup c.3514_3515del	Hom	Belgian	F	Y	Normal	Chorioretinal atrophy	Nystagmus	Occipital meningoencephalocele	Balikova I et al.[4]
c.2969_2978del10 c.1187_1200dup14	Hom	NA	M	NA	NA	NA	Strabismus	Focal alopecia, otitis media and constipation	Thau A et al.[6]
c.2969_2978del c.3514_3515d	Compound het	Brazilian	F	Y	NA	Vitreo-retinal degeneration	None	NA	Suzuki OT et al.[1]

el									
c.2970_2971delAGinsC	Hom	Hispanic	M	Y	Normal	BE: chorioretinal atrophy, RE: RD	Nystagmus	Polymicrogyria	White RJ et al.[7]
c.3213dupC	Hom	NA	F	Y	NA	Choroiretinal atrophy and atrophic macular patches, RD	Nystagmus and esotropia	Bifrontal polymicrogyria	Gradstein L et al.[8]
c.3213dupC	Hom	NA	F	Y	Cortical lens opacity and lens subluxation	Chorioretinal atrophy	None	Unilateral duplex kidney/bifid ureter	Hull S et al.[5]
c.3277C>T	Hom	Brazilian	M	Y	NA	Vitreo-retinal degeneration,retinal detachment	None	Epilepsy	Suzuki OT et al.[1]
c.3356_3357insT	Hom	NA	M	Y	Normal	Chorioretinal atrophy and RD	NA	Midline occipital defect, congenital	Hull S et al.[5]

c.3459dupC	Hom	NA	M	Y	cortical lens opacity and lens subluxation	Chorioretinal atrophy	None	hydronephrosis and hypermobile joints Midline occipital defect and learning difficulties	Hull S et al.[5]
c.3514_3515del c.2105del	Compound het	North American	M	Y	NA	Vitreo-retinal degeneration,retinal detachment	None	NA	Suzuki OT et al.[1]
c.3514_3515delCT	Hom	El Salvadorian	F	Y	NA	Choroiretinal atrophy	None	Occipital dermal sinus tract, frontal cortical dysplasia, white matter loss, mild ventriculomegaly and developmental delay	Mahajan VB [9]

c.3514_3515delCT	Hom	Saudi	M	Y	Ectopia lentis	Vitreo-retinal atrophy	Nystagmus	None	Khan AO et al.[2]
c.3514_3515delCT	Hom	NA	M	NA	NA	NA	Nystagmus	Dermal sinus	Thau A et al.[6]
c.3825_3838del	Hom	Iranian	F	Y	Normal	Chorioretinal atrophy and macula lesion	Nystagmus and esotropia	NA	Haghighi A [10]
c.4054_4055del	Hom	NA	F	Y	NA	RD related to macular hole	Nystagmus	NA	Alsulaiman et al.[3]
c.4063_64delCT	Hom	NA	M	Y	Poor pupillary dilatation	Chorioretinal atrophy and RD	None	Epilepsy and developmental delay	Hull S et al.[5]
c.4063_64 delCT	Hom	NA	F	Y	NA	Chorioretinal atrophy	Glaucoma	Occipital bone defect	Wawrzynski J et al. [11]
c.4173G>A	Hom	NA	F	N	Poor pupillary dilatation	RE:Choroiretinal atrophy, LE:RD and macular hole	None	Occipital encephalocele	Hull S et al.[5]
c.4224_4225delinsC	Hom	Chilean	F	Y	NA	NA	Strabismus	Sinus pericranii	Nakousi-Capurro N et al.[12]
c.4259-28_4265del	Hom	NA	M	Y	Normal	Peripapillary atrophy, leopard fundus and macular atrophy	Strabismus	Scalp defect region and occipital bone defect	Wang P et al.[13]

c.4269_4287del c.4194_4221del	Compound het	Jewish	F	Y	Irregular pupil	Albinotic fundus	None	Normal	Levinger N et al.[14]
c.4290_4299del	Hom	NA	M	Y	Normal	Peripapillary atrophy, leopard fundus and macular atrophy	Strabismus	Scalp defect region	Wang P et al.[13]
c.4492delG	Hom	Belgian	F	Y	Absent iris crypts	BE: Chorioretinal atrophy, LE: RD	NA	Congenital hip subluxation and developmental delay	Balikova I et al.[4]
c.4759_4760del	Hom	NA	M	Y	cataract	Peripapillary atrophy, leopard fundus and macular atrophy	Nystagmus and strabismus	Scalp defect region	Wang P et al.[13]
c.4759_4760delCT	Hom	Chinese	F	Y	cataract	Myopic fundus and macular degeneration	None	Bilateral frontal gyrus dysplasia	Zhang LS et al.[15]
c.4759_4760delCT	Hom	Arab	M	Y	Cataract	Chorioretinal atrophy and macular coloboma	None	Occipital meningocele polymicrogyria	Levinger N et al.[14]

c.4164dupC	Hom	Arab	F	Y	Normal	Chorioretinal atrophy and macular coloboma	None	Atretic meningocele and polymicrogyria	Levinger N et al.[14]

Hom, homozygous; Het, heterozygous; NA, not available; F, female; M, male; Y, yes; BE, both eyes; RE, right eye; LE, left eye; RD, retinal detachment.

1. Suzuki OT, Sertie AL, Der Kaloustian VM, et al. Molecular analysis of collagen XVIII reveals novel mutations, presence of a third isoform, and possible genetic heterogeneity in Knobloch syndrome. *Am J Hum Genet* 2002;*71*(6):1320-9.
2. Khan AO, Aldahmesh MA, Mohamed JY, et al. The distinct ophthalmic phenotype of Knobloch syndrome in children. *Br J Ophthalmol* 2012;*96*(6):890-5.
3. Alsulaiman SM, Al-Abdullah AA, Alakeely A, et al. Macular Hole-Related Retinal Detachment in Children with Knobloch Syndrome. *Ophthalmol Retina* 2020;*4*(5):498-503.
4. Balikova I, Sanak NS, Fanny D, et al. Three cases of molecularly confirmed Knobloch syndrome. *Ophthalmic Genet* 2020;*41*(1):83-7.
5. Hull S, Arno G, Ku CA, et al. Molecular and Clinical Findings in Patients With Knobloch Syndrome. *JAMA Ophthalmol* 2016;*134*(7):753-62.
6. Thau A, Tsukikawa M, Wangtiraumnuay N, et al. Optical Coherence Tomography in Knobloch Syndrome. *Ophthalmic Surg Lasers Imaging Retina* 2019;*50*(8):e203-e10.
7. White RJ, Wang Y, Tang P, Montezuma SR. Knobloch syndrome associated with Polymicrogyria and early onset of retinal detachment: two case reports. *BMC Ophthalmol* 2017;*17*(1):214.
8. Gradstein L, Hansen RM, Cox GF, et al. Progressive retinal degeneration in a girl with Knobloch syndrome who presented with signs of ocular albinism. *Doc Ophthalmol* 2017;*134*(2):135-40.
9. Mahajan VB, Olney AH, Garrett P, et al. Collagen XVIII mutation in Knobloch syndrome with acute lymphoblastic leukemia. *Am J Med Genet A* 2010;*152A*(11):2875-9.
10. Haghighi A, Tiwari A, Piri N, et al. Homozygosity mapping and whole exome sequencing reveal a novel homozygous COL18A1 mutation causing Knobloch syndrome. *PLoS One* 2014;*9*(11):e112747.
11. Wawrzynski J, Than J, Gillam M, Foster PJ. Acute Angle Closure in Knobloch Syndrome. *J Glaucoma* 2021;*30*(5):e265-e8.

12. Nakousi-Capurro N, Huserman J, Castillo S, et al. Knobloch syndrome in a patient from Chile. *Am J Med Genet A* 2020;*182*(10):2239-42.
13. Wang P, Jia X, Xiao X, et al. An Early Diagnostic Clue for COL18A1- and LAMA1-Associated Diseases: High Myopia With Alopecia Areata in the Cranial Midline. *Front Cell Dev Biol* 2021;*9*:644947.
14. Levinger N, Hendler K, Banin E, et al. Variable phenotype of Knobloch syndrome due to biallelic COL18A1 mutations in children. *Eur J Ophthalmol* 2020:1120672120977343.
15. Zhang LS, Li HB, Zeng J, et al. Knobloch syndrome caused by homozygous frameshift mutation of the COL18A1 gene in a Chinese pedigree. *Int J Ophthalmol* 2018;*11*(6):918-22.
